# Multivariate cross-classification: applying machine learning techniques to characterize abstraction in neural representations

**DOI:** 10.3389/fnhum.2015.00151

**Published:** 2015-03-25

**Authors:** Jonas T. Kaplan, Kingson Man, Steven G. Greening

**Affiliations:** ^1^Brain and Creativity Institute, University of Southern CaliforniaLos Angeles, CA, USA; ^2^Department of Psychology, University of Southern CaliforniaLos Angeles, CA, USA; ^3^Department of Gerontology, University of Southern CaliforniaLos Angeles, CA, USA

**Keywords:** MPVA, multivariate pattern analysis techniques, fMRI methods, multivariate pattern classification, multivariate pattern analysis, similarity-based representation

## Abstract

Here we highlight an emerging trend in the use of machine learning classifiers to test for abstraction across patterns of neural activity. When a classifier algorithm is trained on data from one cognitive context, and tested on data from another, conclusions can be drawn about the role of a given brain region in representing information that abstracts across those cognitive contexts. We call this kind of analysis Multivariate Cross-Classification (MVCC), and review several domains where it has recently made an impact. MVCC has been important in establishing correspondences among neural patterns across cognitive domains, including motor-perception matching and cross-sensory matching. It has been used to test for similarity between neural patterns evoked by perception and those generated from memory. Other work has used MVCC to investigate the similarity of representations for semantic categories across different kinds of stimulus presentation, and in the presence of different cognitive demands. We use these examples to demonstrate the power of MVCC as a tool for investigating neural abstraction and discuss some important methodological issues related to its application.

## Introduction

Cognitive neuroimaging has historically been concerned with finding differences. From the early days of neuroimaging, the subtraction technique was employed to identify brain regions where activity differed between one task condition and another, so that we might infer specificity in the function of that region (Posner et al., 1988). More recently, multivariate pattern analysis (MVPA) has become popular partly due to its sensitivity to small differences in activity patterns that univariate techniques are often unable to detect (Haynes and Rees, 2006; Norman et al., 2006; Tong and Pratte, 2012). For instance, MVPA has been used to demonstrate the content-specificity of neural representations within small regions of interest (Kriegeskorte and Bandettini, 2007). However, in addition to this well-deserved reputation as a sensitive difference-detector, there is now a growing appreciation of the power of machine learning techniques to provide evidence for *similarity* among neural patterns.

In an MVPA experiment, a machine-learning classifier algorithm is typically trained on data from a subset of the experiment, and then tested on a held-out set of data that it has not seen before. The classifier will only succeed in predicting the identity of the test trials if learning from the training set transfers to the testing set. Often, a cross-validation procedure is employed where each subset of the data is alternately used as a training and testing set (Pereira and Botvinick, 2011). When a classifier can guess the identity of the testing trials with greater than chance accuracy, we conclude that the data contain information about the class of the stimuli, and that this information is consistent across the various subsets of data.

**Figure 1:**
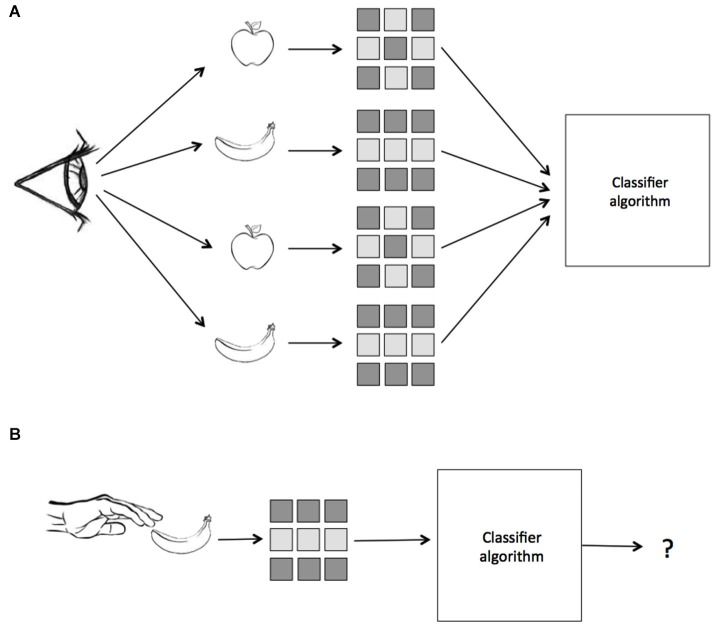
**A schematic of Multivariate Pattern Similarity Analysis**. In this example, subjects either see or touch two classes of objects, apples and bananas. **(A)** First, a classifier is trained on the labeled patterns of neural activity evoked by seeing the two objects. **(B)** Next, the same classifier is given unlabeled data from when the subject touches the same objects and makes a prediction. If the classifier, which was trained on data from vision, can correctly identify the patterns evoked by touch, then we conclude that the representation is modality invariant.

Thus, by requiring learning transfer from training to testing datasets, MVPA constitutes a test for the consistency of information across different sets of data. This property of the test has begun to be exploited by neuroscientists who are interested in how neural patterns may be similar across different kinds of stimulus presentations, sensory modalities, and cognitive contexts. For instance, a classifier trained on data from visual presentation of objects may be asked to then classify neural patterns elicited by tactile presentations of the same objects. The success of learning transfer in such an experiment would provide direct evidence that the neural representations are similar across the two contexts. In the case of this example we are testing whether or not there is a common coding of object identity that is invariant to visual or tactile presentation. We suggest calling this kind of analysis, when a classifier is trained on data from one cognitive domain and tested on data from another, **Multivariate Cross-Classification (MVCC)**. A schematic of MVCC is presented in Figure 1. In this paper we discuss methodological issues relevant to MVCC and review recent work employing this technique in order to demonstrate its power in contributing to the understanding of abstract neural representations.

There are many domains of cognitive neuroscience where the question of abstraction in neural representations is of theoretical importance. We review several of them here in order to highlight the contributions that MVCC can make to these problems. First, we discuss the issue of neural representations that abstract across cognitive modalities, discussing work that uses MVCC to establish the presence of modality-invariant representations in the perceptual and motor domains. Next, we discuss the application of MVCC to research on memory, where there are questions about the extent to which memory and imagery processes re-instantiate patterns that are similar to original perceptions. Finally, we look at work that explores abstract representations within a single sensory modality, to identify neural patterns that represent semantic content similarly across various stimulus formats, and also across cognitive contexts such as those that vary with attention.

## Classification Across Modalities

### Mirror Neurons

One of the most famous recent cases of potential neural abstraction is that of the so-called “mirror neurons”. These neurons, first identified by single unit recordings in the monkey brain, were found to fire when the monkey performs an action himself, and also when he observes someone else performing the same action (Gallese et al., 1996; Rizzolatti and Craighero, 2004). This apparent abstraction of a neural code across agents has fueled speculation about the role of such neurons in empathy, imitation, communication, and a wide range of other social functions (Gallese and Goldman, 1998; Rizzolatti and Craighero, 2004; Decety and Grèzes, 2006; Uddin et al., 2007; Iacoboni, 2009).

The general phenomenon whereby the human motor system responds to action observation has been relatively easy to establish: a range of fMRI studies have shown activation in motor planning areas during action observation (Iacoboni et al., 1999; Grèzes and Decety, 2001; Johnson-Frey et al., 2003), and transcranial magnetic stimulation, or TMS, has demonstrated a lower threshold of excitability over motor cortex when people are observing actions compared to control stimuli (Fadiga et al., 2005). However, to show that regions in frontal and parietal motor cortices represent particular actions in the same way regardless of who the actor is requires more than a demonstration of increased activity when viewing those actions. One way forward on this issue is to attempt to establish a correspondence in the somatotopy of observed and executed actions. For instance, Buccino et al. (2001) showed that observation and execution of actions made with different effectors followed the same pattern of somatotopy in the premotor cortex. Still, these results do not indicate that different actions involving the same effector are represented with specificity across observation and execution. This is a problem perfectly suited to MVCC: if a classifier trained on data from several actions in one modality can discriminate among the same actions from the other modality, this would provide evidence for action-specific representations that share features across the motor and sensory modalities.

Two studies published in 2008 attempted this, with differing results. Dinstein et al. (2008) used MVPA to classify the neural patterns elicited by observing or executing the three stereotyped actions from the game “rock, paper, scissors”. One brain region, the anterior intraparietal sulcus, yielded greater than chance classification within each modality. This provides evidence for action-specific representations of actions performed and actions observed. However, a cross-modal analysis in which a classifier was trained on one modality and tested on the other, failed to achieve greater than chance performance. This opens the possibility that the action-specific representations in anterior intraparietal sulcus are also modality-specific, that is, different patterns of neural activity correspond to executed and observed actions. This dissociation, whereby classification is successful within modalities but not across them, illustrates the kinds of neural architectures that MVCC is able to distinguish. Simply demonstrating that a brain region is activated by two cognitive modalities, or even that it can classify stimuli within each, does not necessarily establish that a brain region contains cross-modal representations that abstract across the modalities. For example, the region may contain different, intermingled sub-populations of neurons that code for each modality separately.

Some neurons in premotor cortex respond not only to the sight of an action but also to its sound (Kohler et al., 2002; Gazzola et al., 2006). Etzel et al. (2008) investigated the crossmodal properties of the motor system by comparing action execution to auditory perception. Participants performed either hand or mouth actions, and listened to the sounds of hand or mouth actions. A classifier was then trained to distinguish within modality, or across modalities by training on data from the auditory condition and testing on data from the execution condition. While several brain regions yielded above-chance performance in one or both modalities, only one brain region, the premotor cortex, was able to classify across modalities. This pattern of results once again demonstrates that a brain region may be active in a content-specific manner within modalities but not necessarily across modalities. However, the success of a cross-modal classifier in this case provides evidence that premotor cortex manifests a common coding for the effector of an action across perception and action.

A series of recent studies by Oosterhof et al. have addressed this issue using MVCC (Oosterhof et al., 2013). Oosterhof et al. (2010) employed a whole brain searchlight approach in which small groups of voxels from locations around the brain are analyzed successively to generate an information map of classifier performance at each location (Kriegeskorte et al., 2006). Unlike previous studies, which typically test spheres of voxels in a brain volume, this group used a surface-based approach to select groups of voxels that are adjacent on the cortical surface. This analysis revealed regions of above chance cross-modal classification in the anterior intraparietal sulcus and also in the lateral occipital cortex. The success of this approach, in contrast to the negative finding of Dinstein et al. (2008) may be attributable to the improved voxel selection method of the surface-based technique.

Thus, distributed neural patterns evoked by motor execution and action observation can be matched using MVCC, although another recent study by Oosterhof et al. (2012a) found that this cross-modal pattern similarity may only hold for actions observed from the 1st person perspective. For example, a classifier trained on performing actions could not predict the observation of actions if they were presented from a 3rd person point of view. This is important because if mirror neurons are really involved in matching between self and other, they should respond similarly when viewing things from the point of view with which we normally see other people. These results are in line with physiological data showing that many neurons in monkey premotor cortex show preference for actions observed from a certain perspective (Caggiano et al., 2011).

The same group performed an MVCC study comparing neural patterns across action execution and mental imagery for actions (Oosterhof et al., 2012b). We deal with the issue of mental imagery in more detail below, but this study serves as an extension of the motor simulation paradigm so it is included in this section. While there is an accumulation of evidence that motor imagery activates the same neural structures as in action execution, pattern classification techniques have the ability to determine the extent to which these patterns are common across performance and imagination. In this study, participants either performed or imagined performing one of two object-directed hand actions. Cross-modal classification was above chance only in the left anterior intraparietal region, confirming the role of this region in abstract representation of actions. Interestingly, classification only worked in one direction: performance was above chance when training on imagery and testing on action, but not when the classifier was trained on action and tested on imagery. This asymmetry between cross-modal classifiers trained and tested in different orderings reflects an unresolved methodological issue in MVCC that we discuss below.

Mirroring mechanisms involve representations of actions that abstract across one sensory domain, either visual or auditory, and the motor domain. In the next section we consider evidence from MVCC for representations that abstract across two different sensory modalities.

### Cross-Modal Sensory Representations

Actions, objects, and events often stimulate multiple sensory modalities simultaneously. This naturally motivates the study of higher order representations that link the different sensory inputs. Such linkages have been studied using the semantic congruency effect, in which brain activity is modulated by the “matching” of stimuli presented in two sensory modalities (e.g., seeing a picture of a dog and then hearing a dog’s bark, vs. seeing a dog and then hearing a cat’s meow). Regions sensitive to multisensory matching would therefore receive information from both sensory modalities. Prior univariate fMRI studies have employed various contrast and adaptation designs to identify sensory convergence (Amedi et al., 2005; Doehrmann and Naumer, 2008). However, a multivariate approach can detect not only that a congruent pair of crossmodal stimuli were presented, but also that representations of specific objects were similar across the modalities. Connecting to our earlier discussion on the link between vision and the somatosensory-motor modality, a multi-voxel correlation analysis found evidence for similar categorical representation of objects when they were seen and when they were touched (Pietrini et al., 2004). It should be noted, however, that this study established similarity using a correlation measure, rather than with a machine learning classifier, as in a strictly defined MVCC. Patterns of activity in inferotemporal cortex were correlated across the visual and tactile presentation of shoes; this was also the case for visual and tactile presentation of bottles, although a correlation was not found for faces. Comparing this study on object representation to the earlier reviewed studies on action representation, we may conclude that visuo-somatosensory abstraction does not take place in one unified location, but rather is organized according to the subject matter being represented.

There are fewer studies on abstraction across the auditory and somatosensory modalities. The aforementioned study by Etzel et al. (2008) found that actions, whether heard or performed, were represented similarly in premotor cortex. We are not aware of MVCC studies showing audio-tactile abstraction of objects. However, a univariate fMRI study demonstrated an audio-haptic congruency effect in left fusiform gyrus and posterior superior temporal sulcus (STS; Kassuba et al., 2013). In those regions, the same objects, touched and then seen, evoked more activity at the single voxel level than touching and then seeing two different objects.

Abstraction across the auditory and visual modalities has been more frequently studied with MVCC. Distinct patterns of activity were found for actions and non-actions, and these patterns were conserved across vision and audition (Ricciardi et al., 2013). Making use of voxels from throughout the brain, including from within the action-observation network, a classifier was trained to distinguish videos on the basis of containing actions or not; the same classifier then successfully distinguished sounds on the basis of containing actions or not. This classification was also successful in the opposite ordering, training on sounds and then testing on videos.

Object representations that abstract across audition and vision were found by our group, and localized to the posterior STS (Man et al., 2012). We trained a classifier to distinguish neural representations of the sounds of six different objects, and then decoded the identities of the same objects presented in silent videos (and* vice versa*, training on videos to decode sounds). Successful crossmodal classification indicated that the object exemplars were distinguishable from each other and also represented similarly, whether seen or heard. A related question is whether object *categories* are represented abstractly across audition and vision. Simanova et al. (2014) presented tools and animals in multiple stimulus formats, auditory (spoken names and nonverbal sounds) as well as visual (printed names and photographs). The study reported successful classification across formats—a classifier trained to distinguish object category based on three of the formats could decode object category from the fourth format—but it is unclear if classification was truly crossmodal, as the training and testing sets were not strictly segregated by sensory modality (in which case a classifier would have been trained on spoken names and sounds, then tested on printed names and photographs).

Akama et al. (2012) studied abstract categorical representations of linguistically presented stimuli. A classifier trained to distinguish animals from tools based on their spoken names was also able to distinguish animals from tools based on their printed names (and *vice versa*). This finding is related to that of Shinkareva et al. (2011), who showed, in the visual modality, that the category of tools or dwellings could be decoded across their pictures and printed names.

Finally, an intriguing MVCC study located supramodal representations of emotions evoked through different sensory modalities (Peelen et al., 2010). The authors showed that emotions evoked specific patterns of activity in posterior STS and medial prefrontal cortex, and these patterns were similar whether they were evoked by the visual modality (viewing emotional faces or body postures) or the auditory modality (hearing emotional expressions).

Through these studies, MVCC is making a major contribution to mapping the convergence of sensory information from different modalities in the brain. MVCC is extending basic MVPA findings of content-specific representations to further characterize those representations as also invariant across modalities.

## Cross-Classification in Memory and Imagery

### Memory Recall

A long held assumption in the memory literature is that the recollection of previously encoded memories involves the re-activation of the same, or similar, patterns of neuronal activity present during the original encoding. Early applications of MVCC to memory, therefore, involved training a classification model on brain activation during the encoding phase of a memory experiment, and later testing the model on data from the recall phase. In an early example using this approach Polyn et al. (2005) tested the contextual reinstatement hypothesis, which in part predicts that neural activity leading up to the moment of recollection of a particular stimulus will increasingly resemble the neural activity observed during the encoding of that stimulus. Testing a neural-network classifier on whole-brain data in an independent free-recall session, they found that classification accuracy for the remembered stimulus indeed increased in the several seconds leading up to verbal recall. In a more recent example from the same group, Johnson et al. (2009) asked the additional question of whether the strength of memory recall is related to the reinstatement of a similar cortical neural response that was present at encoding. They trained a classifier to distinguish between three forms of memory encoding of words. One form involved passive encoding—participants silently read the word—the other two forms involved more elaborate encoding—participants had to imagine how an artist would draw the word or generate multiple uses for the word. They tested the classifier in an independent recall phase in which participants rated how well they recalled the word. They found that classification was significantly higher for words remembered in detail compared to those that participants reported as familiar or unfamiliar, but only when considering words that were initially encoded in an elaborate fashion. This suggests that indeed the strength of memory recall is influenced by cortical reinstatement of a similar neural pattern, at least for elaborately encoded material.

Use of an MVCC approach has not always proved successful in the context of memory. Rissman et al. (2010) sought to determine if the pattern of activation elicited during an implicit recall task was similar to the pattern elicited during an explicit recall task, following a memory encoding session. In this example, in the implicit recall phase, participants made gender judgments of faces that were either “old” (i.e., presented in the encoding phase), or “new” (i.e., novel faces presented only during the recall phase), while in the explicit recall phase, participants made explicit “old” vs. “new” decisions on the faces. They found no significant classification in either direction of this analysis. One possibility is that this lack of effect was due to the classification approach and model used. They used a regularized linear model to perform classification using whole brain data. While this allows for high interpretability of the sensitivity map (in terms of what brain regions were most important for the within-condition classification), it may affect the ability of the classifier to generalize from one condition to the other. The regularized model could end up ignoring voxels that would have been capable of MVCC, but which were not the most sensitive voxels for discriminating within the classes in the training set. In other words, using a regularized linear model is good for high dimensional problems requiring visualization of the sensitivity map of the model, like whole-brain decoding, but it may end up impairing MVCC by de-weighting, or de-valuing, those voxels with cross-condition information. This issue is not specific to regularization however, and may reflect a general issue with feature selection in MVCC. We discuss the issue of feature selection for MVCC further below, and in Figure 3.

**Figure 2:**
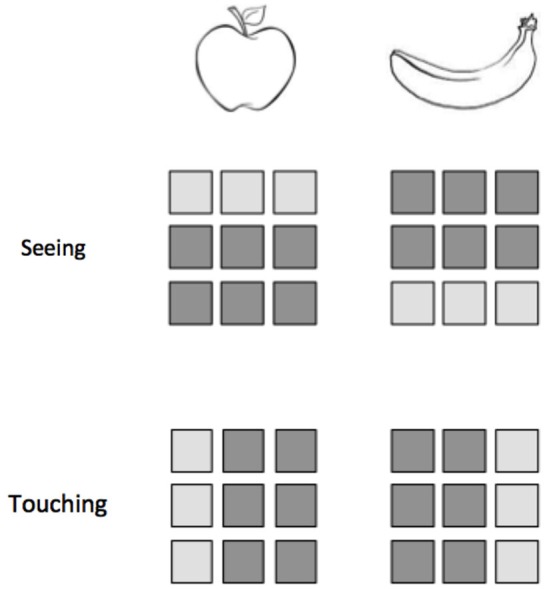
**Classification within modalities does not ensure the success of classification across modalities**. Consider an experiment in which participants either see or touch two objects, an apple and a banana. Here we represent a hypothetical 9-voxel pattern of activity for each stimulus presentation. While the patterns for apple and banana are distinguishable within vision and within touch, a classifier trained on one modality would not be able to correctly identify the patterns from the other modality.

**Figure 3:**
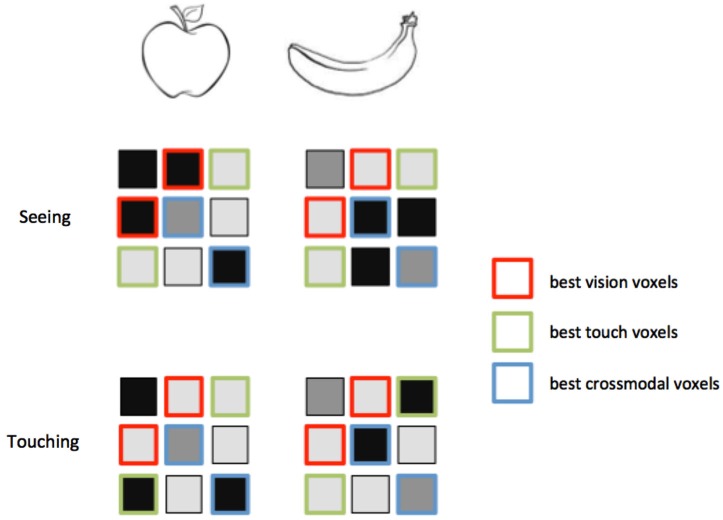
**Feature selection for MVCC**. Voxels that best support intramodal classification are not necessarily the same voxels that support crossmodal classification. Again consider an experiment in which two objects, an apple and a banana, are seen and touched. In these hypothetical activity patterns, the voxels outlined in red distinguish best between the objects when they are seen. However, a different set of voxels, outlined in green, provide the best classification when the objects are touched. Note that the red voxels do not distinguish the objects within touch, and the green voxels do not distinguish the objects within vision. A third set of voxels, outlined in green, provides the best matching between vision and touch. These voxels provide the best crossmodal classification, but do not provide the best intramodal classification. A feature selection technique that only selects the best within-modality voxels might leave out the ones that perform best across modalities.

### Imagery and Imagination

The phenomenon of reinstatement of cortical patterns is also relevant to the study of mental imagery, as of course memory and imagery are intimately linked. Several groups have used MVCC to test the hypothesis that patterns invoked during perception are similar to those invoked by imagination. Much of this work has taken place in the domain of vision. While it has been known for a long time that visual imagery activates visual cortex (Kosslyn et al., 1995), and that the differing contents of visual imagery can activate different regions in visual cortex (Ishai et al., 2000; Mechelli et al., 2004), MVCC provides a more direct test of the specificity of relationship between perceptual and imagery patterns. For instance, Stokes et al. (2009) compared seeing the letters “X” and “O” to imagining the same letters. They found that a classifier trained on data from the lateral occipital complex during the perception condition could correctly classify data from the imagery condition. This result establishes the correspondence between the coding of these two fine-grained visual patterns across perception and imagery. A follow-up study established that the imagination-induced patterns were shape-specific and position-invariant, in that they could be decoded from visual presentations at several locations in the visual field (Stokes et al., 2011).

Reddy et al. (2010) performed a similar experiment using objects from four categories: tools, food, faces, and buildings. Focusing on object-sensitive voxels in the ventral temporal cortex (including lateral occipital complex, fusiform face area, and parahippocampal place area), they found that a support vector machine could classify the four categories across perception and imagery, in either direction. Interestingly, MVCC was not successful in lower-level retinotopic visual regions; the neural similarity between perception and imagery appeared to be restricted to visual regions that represented more abstract object features. This may be a function of the experiment’s focus on categorizing high-level object categories. Indeed, a study which used fine grating patterns to classify across perception, imagery, and working memory, did find successful classification across these conditions in early visual cortex (Albers et al., 2013). Also consistent with this interpretation, Cichy et al. (2012) found that common representations of category across imagery and perception were restricted to high level visual areas, but representations of location were common across seeing and imagining both in high-level and low-level visual areas. Related to imagery, Coutanche and Thompson-Schill (2014) found that top-down, anticipatory visual processing not only resembled visual perception, but also occurred in a feature-specific manner. Cued, but not yet presented, objects could be cross-decoded on the basis of shape (but not color) in lateral occipital cortex, whereas they could be cross-decoded on the basis of color (but not shape) in V4. The re-instantiation of neural patterns in high-level visual cortex during imagery was also corroborated by Johnson and Johnson (2014), who found that a classifier trained on data from perception of complex visual scenes could successfully distinguish among patterns evoked during imagination of the same scenes. Perhaps more impressively Horikawa et al. (2013) found that visual imagery experienced in the early stages of sleep may evoke activity patterns in higher visual cortex that are similar to those evoked by (awake) visual perception of the same types of objects.

## Cross-Classification in Language and Semantic Representations

One domain where the issue of abstract representations in the brain is important concerns the question of how semantic concepts are implemented. For instance, one longstanding assumption is of a correspondence between patterns of brain activity produced by semantically similar words and pictures (Vandenberghe et al., 1996). A study by Shinkareva et al. (2011) employed an MVCC approach to test this assumption. Participants viewed words or pictures belonging to two semantic categories (tools or dwellings), and trained a Gaussian Naïve Bayes classifier on word-evoked activity and then tested it on picture-evoked activity, and* vice versa*. Using voxels selected from throughout the cortex, this cross-classification was significant for both directions, though accuracy was higher when training on word-evoked activity and testing on picture-evoked activation. Furthermore, region-of-interest (ROI) analyses revealed reliable classification in specific regions including inferior parietal lobe, superior parietal lobe, and parts of the extrastriate cortex. By training the classifier using data from all but one subject, and then testing it on the left out subject (leave-one-subject-out cross validation), Shinkareva et al. (2011) also demonstrated significant cross-classification across participants, which is suggestive of semantic similarity that generalizes across individuals. Such findings of generalized semantic representations are further supported by the findings of Quadflieg et al. (2011), in which a classifier training on “up” and “down” shapes can be used to classify words that reflect the concepts of “overhead” vs. “underfoot” concepts.

Another way to study semantic representations is to examine how the same concept, expressed in different languages, may evoke similar neural representations. Two MVCC studies have found evidence for such language invariant representations. Buchweitz et al. (2012) presented the printed names of various objects in Portuguese and in English to bilingual speakers, training a classifier to decode objects named in one language and then testing it to decode the same objects named in the other language. This was successful in both directions of languages used for training and testing, although classifier performance was slightly better when training in Portuguese to test in English. Because the participants were native Portuguese speakers and late learners of English, this suggests that higher classification accuracy may be achieved by training on the modality with stronger or more distinct representations. A related study using spoken words (in Dutch and English) performed whole brain searchlight mapping of cross-language decoding (Correia et al., 2014). Classifiers trained to distinguish neural representations of Dutch words could decode neural representations of English words, and* vice versa*, most prominently in left anterior temporal lobe (lATL) and the right posterior STS. This study, using spoken words, identified a more dorsal region of the lATL than prior studies using written words, hinting at a shift in the location of representation for spoken vs. written linguistic stimuli.

## Classification Across Cognitive Contexts

An important question for cognitive neuroscience is how cognitive contexts, like attention or working memory, influence the neural representation of perceived stimuli. Rather than testing for similarities between two distinct conditions of interest, MVCC can be used to assess how some predefined neural patterns change *within* or *across* competing cognitive contexts. An early example of the former involved the use of MVCC to examine the effects of feature-based attention on perceptual representations of stimulus features. More recent studies of the former involve assessing the similarity of activity patterns during attention vs. working memory for a given stimulus. These two cases will be discussed in turn.

The biased competition theory of attention posits that simultaneously presented visual objects compete for neural representation in a mutually inhibitory fashion. This competition is biased such that the stimulus that is highest in priority, either due to its physical saliency or top-down relevance, “wins” (Bundesen, 1990; Desimone, 1998; Kastner and Ungerleider, 2001; Beck and Kastner, 2009). In feature-based attention, priority is given to one of two overlapping stimulus dimensions. According to biased-competition theory (Desimone and Duncan, 1995; Beck and Kastner, 2009), neural activity corresponding to the representation of the attended feature dimension should be enhanced, and “win” over the unattended dimension. While feature-based attention has been studied using traditional univariate fMRI (Saenz et al., 2002; Schoenfeld et al., 2007), overlapping visual features can pose an issue for the univariate approach. When these overlapping visual features are low-level and similar (i.e., overlapping line orientations), the traditional univariate approach can lack sensitivity (Jehee et al., 2011).

In an early example of MVCC, Kamitani and Tong (2005) demonstrated that feature based attention (i.e., attending to one of two overlapping oriented lines) biased the pattern of brain activity such that they could predict which line was being attended. Specifically, they trained a model to classify differences between two oriented lines (45° and 135°) from trials in which the lines were presented alone. This model was then tested on data from trials in which the two line orientations overlapped. Consistent with predictions of biased-competition, Kamitani and Tong (2005) found that this classifier predicted the attended orientation. This indicates a similarity between the activity patterns evoked by the line presented alone and those evoked by the line presented with another line under the condition of attention. In a follow-up study, Kamitani and Tong (2006) confirmed their earlier finding using overlapping directions of motion. Together these findings demonstrate that feature-based attention modulates the neural patterns that are involved in non-competitive instances of perception (i.e., instances where the oriented lines were presented alone), by making the attended activity pattern more similar to the unambiguous pattern. Serences and Boynton (2007) later found that the influence of feature-based attention spread to ipsilateral and unstimulated regions of visual cortex. However, using MVCC they found that representations were not necessarily shared across the two types of attention, feature-based and spatially-spread. They trained a classifier to predict the direction of attended-motion in a region of V1 corresponding to the region of retinotopic stimulation (i.e., the contralateral hemifield). They then tested the classifier on the same set of voxels in V1 but on trials in which visual stimulation occurred in the ipsilateral hemifield, and found non-significant classification.

While the work described above demonstrates the use of MVCC in elucidating the influence a given cognitive process (e.g., feature-based attention) has on patterns of neural activity, more recent work has extended this approach to examine whether two distinct cognitive processes work by modulating similar patterns of neural activity. For example, recent research has demonstrated that both attention and visual working memory influence similar perceptual representations to a significant extent. Serences et al. (2009) demonstrated that the pattern of activity evoked when attending to a visual stimulus is similar to the pattern evoked during visual working memory maintenance of the same stimulus in the absence of retinal input. Using an MVCC approach, Serences et al. (2009) trained separate models to predict when participants were attending to either the color (i.e., green/red) or line orientation (45° or 135°) while ignoring the other dimension. They tested the model on separate runs in which participants were asked to maintain either the color or line orientation of the stimulus in working memory in the absence of visual stimulation. Averaging across these two analyses they found above chance classification in primary visual cortex (V1). In a later example, Ester et al. (2009) found a similar effect using MVCC on patterns of activity in non-stimulated regions of retinotopically mapped V1. Notably, in neither of these two examples did the authors perform the reverse analysis in which a model is trained on the visual working memory data and tested on the visual attention data (see below for a discussion of directionality in MVCC). Using a similar approach, Harrison and Tong (2009) found that even when oriented lines were presented but unattended, the pattern of activity in this context was similar to the pattern of activity evoked during visual working memory maintenance of the same oriented lines, and* vice versa*. However, the accuracy for both directions of this analysis were averaged, making it unclear whether there are accuracy differences when moving in one direction vs. the other (i.e., training on visual stimulation trials and testing on visual working memory trials might produce better results than training on visual working memory trials and testing on visual stimulation trials). Nevertheless, together the above examples demonstrate how a pattern of neural activity corresponding to a specific stimulus can be modulated by distinct cognitive process (i.e., across cognitive contexts) and the utility of MVCC in testing such predictions.

## Relation to Other Approaches

There are several related neuroimaging techniques that can contribute to understanding how neural representations relate to one another across contexts. For instance, one of the earliest approaches to answering similar questions was to employ univariate localizers. A localizer task can be used to identify a region of the brain that has certain response properties, and that region can be subsequently tested in a new task. This is roughly analogous to MVCC in that the experiment is divided into two parts, one to identify a functional ROI based on known or expected properties, and another to test something new about that ROIs response (Saxe et al., 2006). For instance, consider an experiment in which a region of somatosensory cortex is identified by contrasting touches of the hand compared with touches of the foot. Next, this region is tested for its response when observing touches of someone else’s hand vs. someone else’s foot. If the univariate response in the ROI is greater both for observation of touch and experience of touch, it can be argued that the two tasks share a common substrate.

However, the univariate localizer approach has limitations. Univariate analysis relies on spatial smoothing and is insensitive to fine-grained spatial patterns which may carry information (Haynes and Rees, 2006; Kriegeskorte et al., 2006; Norman et al., 2006). Thus, while the univariate localizer approach can establish a broad correspondence across tasks regarding overall levels of activation within an ROI, it is not sensitive to cases where information is represented in distributed codes across populations of neurons. Similar univariate activation across two tasks may belie more subtle differences, and the literature is replete with examples in which activation across tasks does not coincide with differences in content-specific information as revealed by MVPA (see, for example, Woo et al., 2014). To use an example from our own work (Man et al., 2012), univariate activation in response to seeing and hearing objects revealed several regions of overlap across the two sensory modalities. However, only one of these brain regions, the posterior STS, displayed both content-specificity and modality-invariance as revealed by MVCC analysis. It is important to note that since MVPA is sensitive to both global and distributed activity differences, successful MVPA classification does not imply the presence of a distributed representation (Davis and Poldrack, 2013; Davis et al., 2014a).

Among multivariate approaches to analyzing the similarity of neural representations, there are generally two levels of analysis: those made in *voxel-activity space*, and those made in *representational space* (Haxby et al., 2014). In the first kind of analysis, voxel activity patterns across two different domains are directly compared. MVCC falls into this category, but there are other, similar approaches. For example, one can compute a distance metric between two vectors of voxel activations that represent the responses to two stimuli in different tasks. This metric is commonly a simple correlation between the vectors, or alternately a measure of Euclidean distance in n-dimensional space, where n is the number of voxels. In the example of our cross-sensory task (Man et al., 2012), we could compare the correlation between seeing a bell and hearing a bell to the correlation between seeing a bell and hearing a typewriter. Increased similarity among responses to the same object type provides evidence for cross-modal invariance. These distance metrics can be quantitatively analyzed (Pietrini et al., 2004; Ritchey et al., 2013; Davis et al., 2014b) or may become the basis for a minimum distance classifier (Mur et al., 2009), as was used by Haxby et al. (2001) and Spiridon and Kanwisher (2002).

In the second kind of analysis, the measured activity patterns are translated into an abstract format that represents the relationships among the voxel activity patterns for different stimuli. Comparisons are then made in the constructed representational space. This approach is known as Representational Similarity Analysis (RSA; Kriegeskorte et al., 2008a; Kriegeskorte and Kievit, 2013). In RSA a distance metric is computed among all pairs of stimuli which yields a matrix called a representational dissimilarity matrix (RDM) that can then be compared across very different contexts, such as across different imaging modalities, different individuals, or even different species. In a powerful example of this approach Kriegeskorte et al. (2008b) show that the dissimilarity matrices for object representations in inferotemporal cortex correspond across human and monkey brains. While this approach shows that the internal relationships among representations of object types are common across the species, it does not show commonality in the actual neural patterns used to represent those objects across species. For example, in both a human and a monkey, patterns evoked by bananas are more similar to patterns evoked by carrots than they are to patterns evoked by faces. However, the neural patterns used to represent a banana in a human brain might be very different from those in a monkey brain. Indeed, voxelwise patterns would be difficult or impossible to compare given the differences in anatomy between the two species. The RSA approach seeks to solve this problem by comparing higher-order relationships among the patterns evoked by the set of objects instead of comparing the activity patterns directly. Of course RSA can be used within species and within individuals to compare representational spaces evoked by different tasks. But in cases where voxels can be expected to correspond across domains, the MVCC approach has the power to directly compare activity patterns without the intermediary step of an abstracted RDM.

## Methodological and Interpretational Considerations

There are several methodological questions that come into play regarding the details of performing MVCC. For instance, when training a classifier on one stimulus set and testing on another, the issue of training direction, or ordering, may be important. Which stimulus set should serve as the training data and which as the testing data? Some papers report classification results averaged across both directions of training (Man et al., 2012; Oosterhof et al., 2012b), some report only a single direction (Etzel et al., 2008; Johnson and Johnson, 2014), and some report both directions separately (Quadflieg et al., 2011; Akama et al., 2012). Sometimes there are theoretical reasons that motivate favoring one direction of classification over another. As an example, Etzel et al. (2008), in studying auditory mirror neurons, reasoned that the neurons with auditory properties constituted only a subset of the neurons within their ROI. Only the information from this subset of neurons should be expected to transfer to the motor modality; therefore they trained on auditory stimulation and tested on motor stimulation.

In the absence of such a theoretical motivation, however, we might consider more generally how intramodal classification relates to crossmodal classification (see Figure 2). One open question concerns whether it is better to train or test on the modality where the data are less noisy and the patterns are more easily distinguished. Consider again a visual-tactile MVCC in visual cortex. We can expect the patterns evoked by vision to be more separable and reliable than those evoked by tactile stimulation. In this circumstance, does a classifier trained on vision and tested on tactile outperform a classifier trained on tactile then tested on vision? Until answers to these questions become clarified, we recommend reporting both directions of classification in order to avoid “cherry picking” the best results.

The question of classification direction is closely related to the issue of feature selection. The voxels that best distinguish the classes within one modality are not necessarily the same voxels that contain the best modality-invariant information (see Figure 3 for a graphical depiction of this idea). For this reason, it may be better to choose voxels based on a statistic that reflects activation in both modalities rather than just one modality. A region that represents both modalities in an invariant manner would presumably become activated by either modality presented in isolation. An MVCC study may include a separate functional localizer that identifies voxels strongly co-activated by both modalities. This provides an independent dataset for voxel selection in a subsequent MVCC analysis.

Somewhat counterintuitively, we argue that it may be valid to perform voxel selection using information from the testing set and still avoid circular analysis, in special cases of MVCC. Circular analysis is defined as using the same data for selection as for selective analysis (Kriegeskorte et al., 2009). An example of circularity may be seen in *intramodal* classification: voxel selection is performed with a searchlight analysis of the entire dataset, and then the same dataset is split into training and testing sets to assess classification accuracy in the most sensitive voxels, as determined by the searchlight. In this example, the voxels are selected partly due to their good performance on examples within the testing set, so performance of this intramodal classifier will be inflated. A crossmodal classifier, however, specifically tests a cross-generalization hypothesis that representations are similar across modalities. Within-modality success does not imply cross-modality success (see Horikawa et al., 2013 for a similar argument). Selection of voxels that decode stimuli accurately in either modality does not bias the null distribution of generalization accuracies. Applying the policy outlined in Kriegeskorte et al. (2009) prevents circularity under these selection procedures by modeling the effect of selection under the null. This will typically be performed with a permutation test that scrambles the labels of stimuli in both modalities, performs an identical selection procedure (e.g., running a searchlight for each modality, then selecting voxels that were highly accurate in both searchlights), then performing the crossmodal classification to yield a null accuracy value. This procedure is repeated to form a null distribution that models the effect of selection.

It is also important to note that performance on a cross-modal classification may be constrained by intra-modal classification performance. In particular, the accuracy of intra-modal classifiers may set the upper bound of what we should expect for a cross-modal classifier. We would not expect a cross-modal classifier to perform significantly higher than the accuracy of the best intra-modal classifier. Crossmodal accuracies in MVCC should therefore be considered in the context of the corresponding intramodal accuracies, the best of which may serve as a “soft ceiling” for expected crossmodal performance.

While an MVCC analysis can provide positive evidence for the invariance of representations across contexts, it is important to keep in mind that successful learning transfer from one modality to another may have alternate explanations. The presence of a confound that co-occurs with conditions across modalities could produce successful cross-decoding. This is actually a specific case of a more general issue that affects MVPA studies (Todd et al., 2013). For instance, consider a cross-modal MVCC study in which participants see and hear two objects. If one object draws more attention when seen and heard, then a classifier may distinguish the voxels that respond to the objects across vision and hearing only because of this attentional difference and not because of an underlying crossmodal object representation. These kinds of alternate explanations may be less convincing in the case where there are more than two classes that differ along multiple dimensions, but this observation underscores the need for carefully controlled stimuli.

In addition, there are many open methodological questions for MVCC analysis. These include whether certain kinds of classifiers tend to perform better than others in cross-classification contexts, or how preprocessing steps like spatial smoothing affect performance. While these issues have been explored to some extent with regards to MVPA in general (Misaki et al., 2010; Etzel et al., 2011), there are considerations specific to MVCC that should be evaluated. For instance, smoothing may become relevant if neural units that carry information in one modality are intermingled with units that carry information in another modality. Future research is needed to address these questions.

## Conclusions

MVCC is an extension of traditional MVPA that allows comparison of neural patterns evoked by different contexts. By training a classifier on data from one context and testing on another, it is possible to provide evidence for the invariance of neural representations across those contexts. MVCC is evolving into an important tool for cognitive neuroscientists, which has been instrumental in making progress in many theoretical domains. Studies using this technique have contributed to understanding how sensory and motor information is combined, to testing theoretical questions about how memory, imagery, and language are implemented in the brain, and to characterizing the effects of attention and working memory on perceptual representations.

## Conflict of Interest Statement

The authors declare that the research was conducted in the absence of any commercial or financial relationships that could be construed as a potential conflict of interest.
